# Expression of immune checkpoint regulators, programmed death-ligand 1 (PD-L1/PD-1), cytotoxic T lymphocyte antigen 4 (CTLA-4), and indolaimine-2, 3-deoxygenase (IDO) in uterine mesenchymal tumors

**DOI:** 10.1186/s13000-022-01251-2

**Published:** 2022-09-14

**Authors:** Alireza Samiei, David W. Gjertson, Sanaz Memarzadeh, Gottfried E. Konecny, Neda A. Moatamed

**Affiliations:** 1 Department of Pathology and Laboratory Medicine, David Geffen School of Medicine at UCLA, 10833 Le Conte Avenue, 13-145 CHS, BOX 951732, Los Angeles, CA 90095-1732 USA; 2 Department of Biostatistics, Fielding School of Public Health at UCLA, Los Angeles, CA USA; 3 Department of Obstetrics and Gynecology, David Geffen School of Medicine at UCLA, Los Angeles, CA USA; 4 UCLA Eli and Edythe Broad Center of Regenerative Medicine and Stem Cell Research, University of California Los Angeles, Los Angeles, CA USA; 5 Johnson Comprehensive Cancer Center, University of California Los Angeles, Los Angeles, CA USA; 6 Molecular Biology Institute, University of California Los Angeles, Los Angeles, CA USA; 7 VA Greater Los Angeles Healthcare System, Los Angeles, CA USA; 8 Department of Hematology-Oncology, David Geffen School of Medicine at UCLA, Los Angeles, CA USA

## Abstract

**Background:**

Immune checkpoints including programmed death-ligand 1/programmed death-1/ (PD-L1/PD-1), cytotoxic T lymphocyte antigen 4 (CTLA-4), and indolaimine-2, 3-deoxygenase (IDO) have recently emerged as effective candidates for treatment against a range of human malignancies. We have investigated their expression in the uterine mesenchymal tumors.

**Methods:**

Sixty-eight mesenchymal tumors were categorized into 6 diagnostic groups. We assessed PD-L1, PD-1, CTLA-4, and IDO expression on paraffin embedded tissue blocks of the uterine tumors using the respective antibodies. Immunohistochemical (IHC) stains were classified as positive when the reactions were present in at least 1% of the cell membranes for PD-L1/PD-1 or in cytoplasm for CTLA-4 and IDO, regardless of intensity. Student’s t-test and McNemar’s chi-square tests were carried out to analyze the results.

**Results:**

The mesenchymal neoplasms had expressed the immune checkpoints in the tumor and/or the lymphoid cells at the rate of 49% and 54% respectively. The tumor cells were positive in 10 (18%, PD-L1), 0 (0%, PD-1), 18 (32%, CTLA-4), and 13 (23%, IDO) cases while the infiltrating lymphoid cells were positive in 10 (18%, PD-L1), 23 (40%, PD-1), 18 (32%, CTLA-4), and 13 (23%, IDO) cases. Overall, comparison of paired tumor vs lymphoid cells resulted in *p*-values of ≤ 0.04.

**Conclusions:**

Nearly 50% of the uterine tumors express at least one of the immune checkpoints in tumor and/or the infiltrating lymphoid cells. However, expression of the proteins in the two cellular components are mutually exclusive. Namely, when tumor cells express an immune checkpoint, the infiltrating lymphoid cells do not, and vice versa. Since the leiomyosarcomas are reportedly resistant to the immunotherapy when PD-L1 is expressed in the tumor cells, it can be posited that presence of the IHC positive lymphoid cells may be a better indicator of response to the treatment.

**Supplementary Information:**

The online version contains supplementary material available at 10.1186/s13000-022-01251-2.

## Background

Mesenchymal tumors are a heterogeneous group of neoplasms that can occur in the uterus. Majority of these tumors are of uterine smooth muscle and endometrial stromal cells origin, with a ranging spectrum of behavior [1]. Endometrial mesenchymal tumors account for less than 3 percent of all uterine malignant neoplasms and commonly present in patients over the age of 40 [2].

Current therapeutic approaches for these uterine sarcomas include surgery, adjuvant chemotherapy, radiation therapy as well as endocrine therapy [2]. Unfortunately, treatment options are limited when the sarcomas relapse or metastasize. Recently, immune checkpoint inhibitors targeting programmed death-ligand 1/ programmed death- 1 (PD-L1/PD-1) and cytotoxic T lymphocyte antigen 4 (CTLA-4) with or without inhibitors of the immunosuppressive molecule indolamine-2, 3-deoxygenase (IDO), are being explored in the treatment of a number of human malignancies including uterine mesenchymal tumors [3–6]. The patients’ immune system can recognize tumor-specific molecules that allow it to attack the malignant cells. Malignant cells, in turn, can express a variety of check-point proteins that allow the tumor to evade or suppress this immune response. Immune checkpoint therapeutics are designed to amplify the body’s immune response against such tumor cells [7]. For instance, PD-L1, a protein expressed on tumor cells, and PD-1 expressed on tumor-infiltrating immune cells enable the tumor to dysregulate the body’s immune response [7]. Anti-PD-1/PD-L1 antibodies have provided means to counter this tumor-induced immune dysregulation [7].

The immune regulatory enzyme IDO suppresses T-cell responses and may reduce efficacy of therapies targeting immune checkpoint proteins such as described earlier. Early phase clinical trials combining IDO and PD-1/ PD-L1 inhibitors have shown some promise in non-small cell lung cancers (NSCLCs) [8]. CTLA-4 immune checkpoint molecule has been shown to be a negative regulator of T-cell immune function [9–11]. Studies have also demonstrated that CTLA-4 is constitutively expressed on tumor cell lines at various degrees of intensity and can trigger apoptosis of CTLA-4-expressing tumor cells after interaction with soluble CD80 or CD86 recombinant ligands [12]. CTLA-4 has been implicated in immune dysregulation of various solid tumors including esophageal cancer [13], breast cancer [14], lung cancer [15], melanoma [16], non-melanoma skin cancers [17], and cervical cancers [18]. Currently, a clear role for the immune-checkpoint regulators in uterine mesenchymal tumors remain unknown.

The aim of this study was to examine the expression of the immune check-point proteins in uterine mesenchymal neoplasms, to help direct the future clinical development of novel immune check-point inhibitor therapies. Our findings provide a foundation for screening these mesenchymal tumors to identify candidates for the targeted treatments.

## Materials and methods

### Patients’ selection

A computer search of our departmental database (software: Epic Beaker, Atlanta, Georgia) was carried out to obtain a list of the patients diagnosed with uterine leiomyosarcoma (LMS), recurrent and metastatic LMS, endometrial stromal sarcoma (ESS), and smooth muscle tumors of uncertain malignant potential (STUMP) from 2005 to 2020. Also, most recent cases with leiomyomas were randomly selected for the control group. Clinical follow-up status of each individual case was obtained from the electronic medical record when available. This study was reviewed and approved by the Institutional Review Board at David Geffen School of Medicine at UCLA (IRB # 15–001,035-CR-00001).

For this study, all cases were reviewed again to confirm the original histologic diagnoses based on the criteria published in the 2020 World Health Organization Female Genital Tumours [19–23].

### Histopathology and immunohistochemistry

#### Tissue section processing

Formalin-fixed paraffin-embedded tissue sections were used for hematoxylin and eosin (H&E) and immunohistochemistry (IHC) stains. For all antibodies, slides were baked at 60° C for 1 h, then deparaffinized through xylenes and graded ethyl alcohols. Then, they were processed for antigen retrieval with high pH Epitope Retrieval solution (Leica Bond ER2) in a pressure cooker (Biocare Medical, Pacheco, CA) for 5 min, followed by 15 min of cool down before rinsing them in distilled water.

#### Primary antibody incubation and detection

**Fig. 1:**
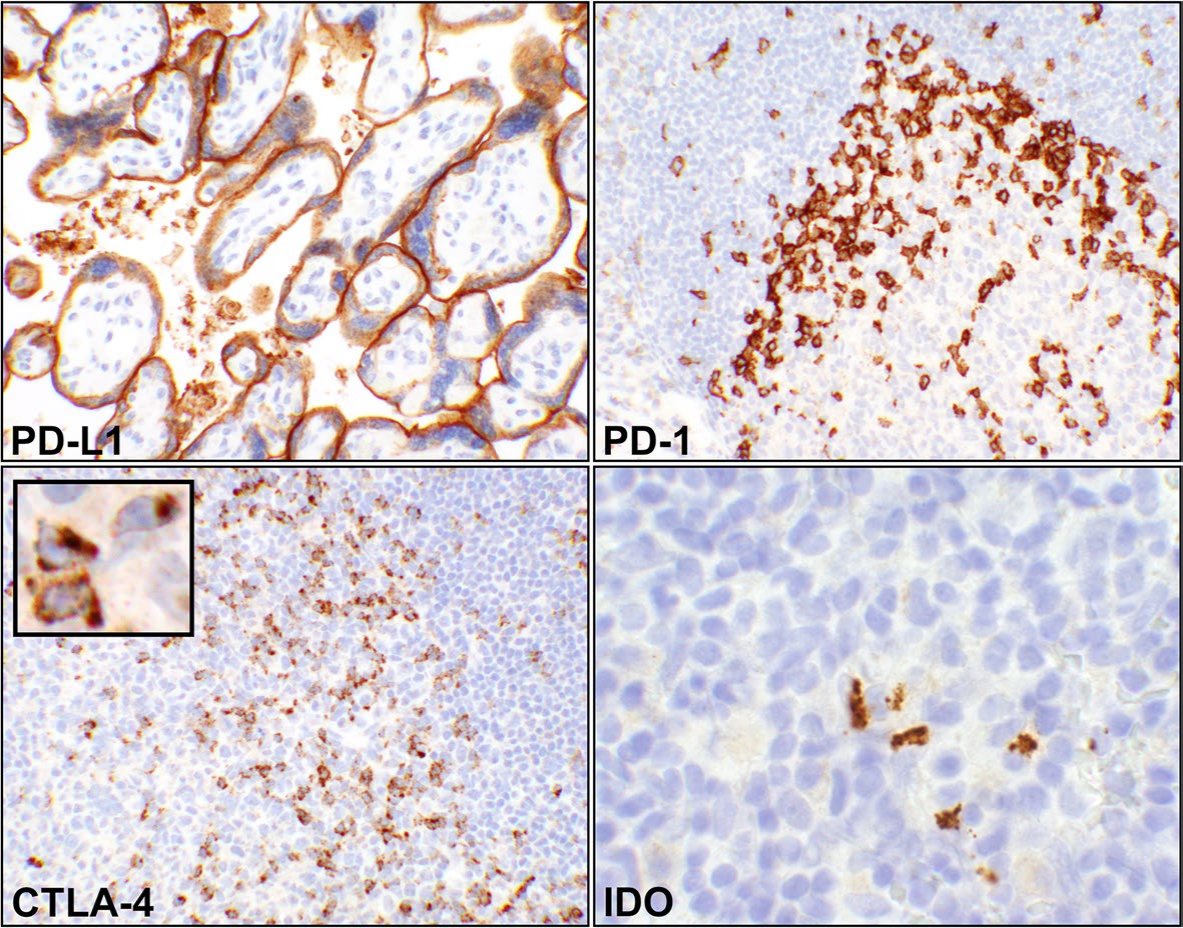
Controls for the immunohistochemical (IHC) reactions. (PD-L1) A photomicrograph showing intense predominant staining of syncytiotrophoblasts appearing as a linear reaction. Tonsils can be also used as control for this immune checkpoint (20 × objective). (PD-1) A portion a lymphoid follicle in tonsil with adjacent germinal center depicting T-helper cells by IHC (20 × objective). The reaction is on cell membrane of the cells. (CTLA-4), Photomicrograph of a segment of a tonsillar germinal center with the adjacent follicular cell (20 × objective). IHC has detected the activated T-cells where the reactions are coarsely granular and intracytoplasmic (inset, 60 × objective, digitally magnified). (IDO) A focus of tonsillar lymphoid tissue showing a few dendritic cells with granular intracytoplasmic reaction (60 × objective). Abbreviations: PD-L1, programmed death-ligand 1; PD-1, programmed cell death protein 1; CTLA-4, cytotoxic T lymphocyte antigen 4; IDO, indolaimine-2, 3-deoxygenase

The histological sections were incubated with the individual antibodies using appropriate dilutions and timing (see below) followed by post-primary antibody for 8 min. Following antibody incubation, using Leica Bond Refine DAB Detection reagents on Leica Bond III Auto-Stainer, the sections were incubated with BOND polymer (Leica, Product# DS9800) for 8 min, followed by post-polymer hydrogen peroxide and diaminobenzidine (DAB) and finally treated with post-DAB cupric sulfate solutions. For controls, tonsil tissue was used for all markers in addition to normal placenta for PD-L1 (Fig. 1).

#### PD-L1

Rabbit monoclonal anti-PD-L1, clone 22C3 (Agilent Dako, Santa Clara, CA), was used at 1/200 dilution and 60 min incubation [24]. Normal human placenta and human tonsil tissues were used as control. The protein is an inhibitory ligand expressed on the surface of the T-cells, dendritic cells, macrophages, and fibroblasts [25, 26]. In human placenta, it is predominantly expressed in syncytiotrophoblasts (Fig. 1) [27].

#### PD-1

Mouse monoclonal anti-PD-1, clone NAT105 (Cell Marque, Rocklin, CA) was used at 1/50 dilution for 30 min incubation. Human tonsil tissue was used as control. PD-1 is a cell membrane anchored molecule that is a marker of germinal center T-helper cells (Fig. 1) [28, 29].

#### CTLA-4

Mouse monoclonal anti-CTLA-4, clone F8 (Santa Cruz Biotech, Santa Cruz, CA) was used at 1/800 dilution for overnight incubation in a refrigerator. CTLA-4, a coreceptor with sequence homology to CD28, is expressed on activated T cells mainly in the germinal centers and T-cell zones (Fig. 1) [30, 31].

#### IDO

Rabbit polyclonal anti-IDO-1 (Abcam, Cambridge​, MA) was used at 1/50 dilution for overnight refrigerated incubation. Human tonsil was used as control. In lymphoid tissues, IDO is expressed in mature dendritic cells with a phenotype distinct from plasmacytoid dendritic cells (Fig. 1) [32].

### IHC evaluation and interpretation

**Fig. 2:**
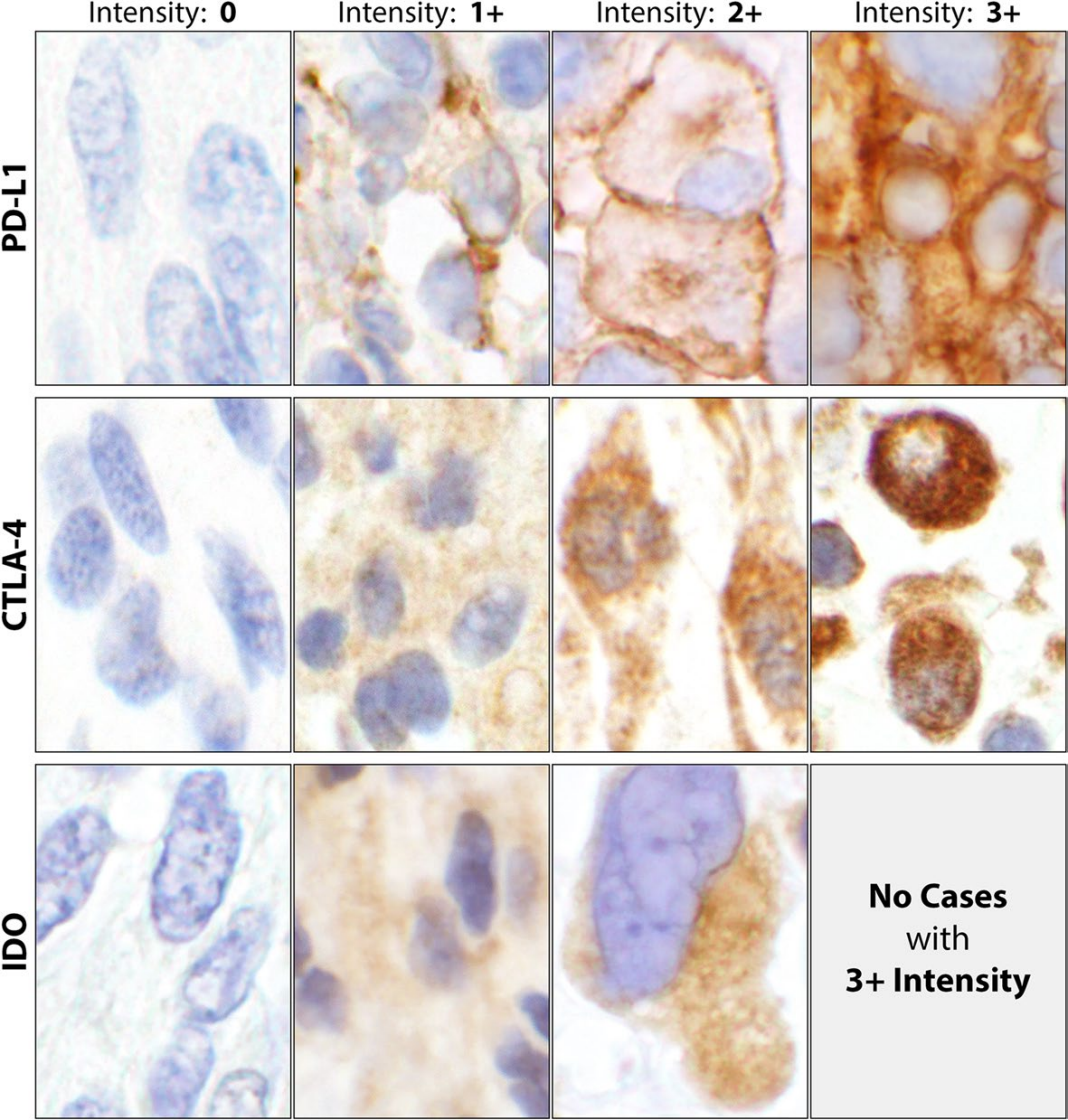
Immunohistochemical (IHC) reactions’ intensities. Intensity 0, showing no cell membrane staining for PD-L1 and no cytoplasmic reactions for CTLA-4 and IDO. Intensity 1 + , showing a weak partial cell membrane staining for PD-L1 and a low number of reactive cytoplasmic granules for CTLA-4 and IDO. Intensity 2 + , the cell membrane staining is more intense and completely circumferential for PD-L1. The IHC reactions depicted a moderate number of reactive cytoplasmic granules for CTLA-4 and IDO. Intensity 3 + , showing a very intense and completely circumferential membrane staining for PD-L1 and a high number of reactive granules packed in the cytoplasm for CTLA-4. Since no 3 + intensity for IDO was encountered in this study, no photograph was included. Generally, the IDO reactions were rather defuse and less granular than CTLA-4. Since no tumor cell staining was noticed, PD-1 was excluded in this Fig. (60 × objective). Abbreviations: PD-L1, programmed death-ligand 1; CTLA-4, cytotoxic T lymphocyte antigen 4; IDO, indolaimine-2, 3-deoxygenase

Although a combined scoring of the tumor and the corresponding lymphoid cells reactions (CPS) is recommended for an improved immunotherapy correlation using PD-L1 [33, 34], De Marchi et al. have shown CPS and tumor positive scoring (TPS) are in full agreement in non-small cell lung cancer tissue specimens [35]. Briefly the CPS scoring is summing the tumor and lymphoid positivity percentages [35]. In this study, however, we have kept the scoring of the reactions for tumor cells (TCs) versus lymphoid cells (LCs) separate as recorded in the Additional tables. PD-L1 and PD-1 expressions were assessed as cell membrane staining while cytoplasmic stains were used for CTLA-4 and IDO (Fig. 2). The recorded results were based on the intensity of staining reaction on the cell membrane for PD-L1/PD-1 or cytoplasm for CTLA-4 and IDO as previously described for the cervical and breast tissues [36, 37]. The scoring system was based on the initial Garon et al.’s study for non-small-cell lung cancers [38], which is briefly summarized below:

- *Intensity 0*: If there was no reaction on cell membrane, or in cytoplasm.
- *Intensity 1* + : If a weak intensity and/or incomplete circumferential cell membrane staining, or a low number of cytoplasmic granules had the reactions.
- *Intensity 2* + : If a complete circumferential cell membrane staining, or a moderate number of cytoplasmic granules had the reaction.
- *Intensity 3* + : If a strong circumferential cell membrane staining, or a high number of cytoplasmic granules had the reaction (Fig. 2).

For this study, any positive reaction of 1% or more was scored as “*positive”* regardless of intensity of the reaction on tumor and/or lymphoid cells [39, 40].

### Statistical analyses

Student’s T-test was employed to assess associations of the immune checkpoints’ reactions in the tumors as well as the infiltrating lymphoid cells with positive versus negative staining. Also, McNemar’s chi-square test was employed to measure the significance of the differences between the correlated proportions of the matched-pair samples [41, 42]. To rejection the null hypothesis, a *p*-value of 0.05 or less was used to indicate a significant difference between the two sets of data. A *p*-value of > 0.05 to < 0.1 was considered as borderline significance in this study. Microsoft Excel was used to tabulate the data, and STATA (Version 17, StataCorp LLC, College Station, TX) was used to perform statistical analyses. Since this study was observational where the number of cases was based on the available data, no formal power calculations were performed. Further, *p*-values were not adjusted for multiplicity due to the study’s exploratory nature. Finally, all subjects with associated tissue samples were selected for study during the defined period.

### Study design

The two pathologists (AS & NAM) reviewed the cases for both accuracy of the diagnoses and the scoring of the IHC reactions. Based on the histopathological diagnoses and the clinical progression, the samples were categorized into six groups: Group I, leiomyoma (as control); Group II, leiomyosarcoma; Group III, recurrent leiomyosarcoma; Group IV; metastatic leiomyosarcoma; Group V, endometrial stromal sarcoma; and Group VI smooth muscle tumors of uncertain malignant potential. The metastatic and recurrent LMSs were grouped separately to assess possible differences in the expression of the immune checkpoints. Tumor and lymphoid cells were evaluated separately for the expression of the immune checkpoints and data were tabulated and summarized for each of the 6 groups. In addition to tabulation of IHC reactions for each individual immune checkpoint, a column was created for combined IHC reactions where the case would have been designated as “positive” if one or more immune checkpoints were expressed (Additional Table 1). Subsequently, a binary table of negative (0) and positive (1) was generated for statistical analyses (Additional Table 2). Also, groups II-IV (all leiomyosarcomas) and II-VI (sarcomas + STUMP) were examined separately as “combined groups” (Tables 1, 2, 3 and 4).

**Table 1:**
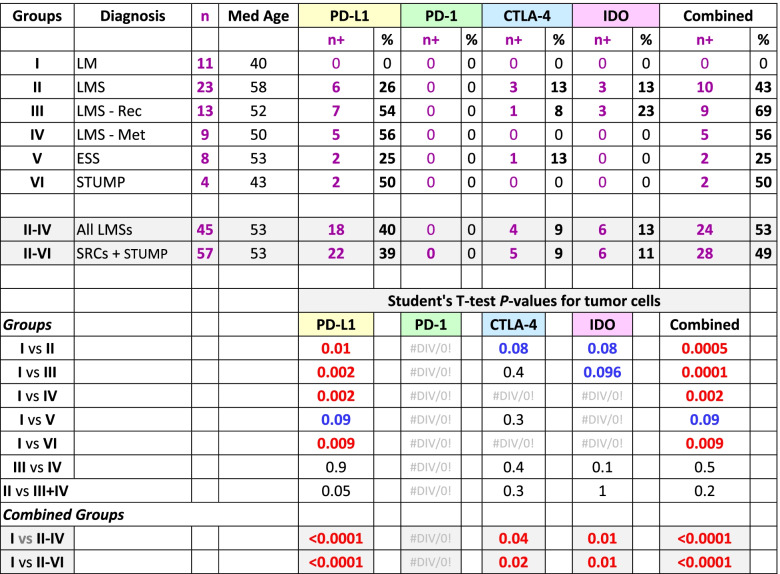
Summary of the results of immunohistochemical reactions of the immune checkpoints on *tumor cells* in each of the six groups of the uterine mesenchymal neoplasms

*LM* Leiomyoma, *LMS* Leiomyosarcoma, *LMS—Rec* Recurrent leiomyosarcoma, *LMS—Met* Metastatic leiomyosarcoma, *ESS* Endometrial stromal sarcoma, *STUMP* Smooth muscle tumor of uncertain malignant potential, *SRC* Sarcoma, *Med* Median, *PD-L1* Programmed death-ligand 1, *PD-1* Programmed cell death protein 1, *CTLA-4* Cytotoxic T-lymphocyte-associated protein 4, *IDO* Indoleamine 2,3-dioxygenase, *n +* Number of positive cases, *+* Positive; Combined n + , at least one of the immune checkpoints is positive; Red, significant difference (*p*-value ≤ 0.05); Blue, borderline significant difference (0.05 < *p*-value < 0.1)

**Table 2:**
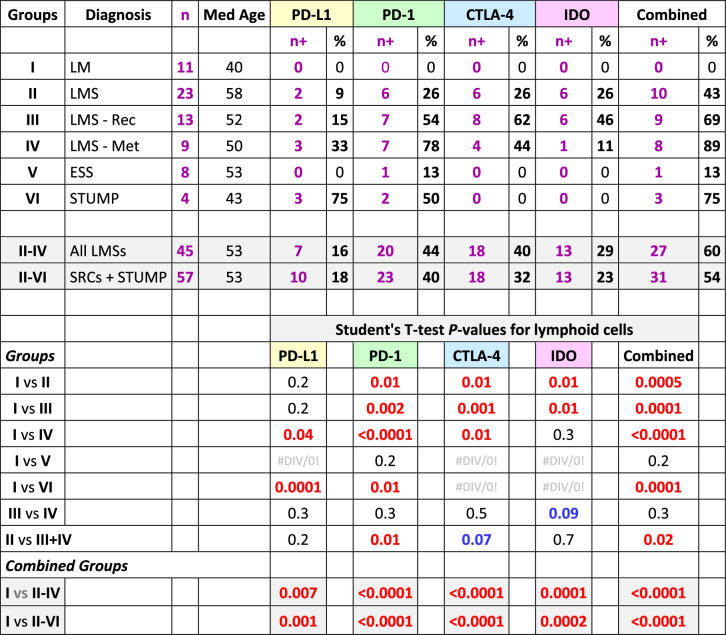
Summary of the immunohistochemical reactions of the immune checkpoints results on the tumor *lymphoid cells* infiltrate in the six groups of the uterine mesenchymal neoplasia

*LM* Leiomyoma, *LMS* Leiomyosarcoma, *LMS—Rec* Recurrent leiomyosarcoma, *LMS—Met* Metastatic leiomyosarcoma, *ESS* Endometrial stromal sarcoma, *STUMP* Smooth muscle tumor of uncertain malignant potential, *SRC* Sarcoma, *Med* Median, *PD-L1* Programmed death-ligand 1, *PD-1* Programmed cell death protein 1, *CTLA-4* Cytotoxic T-lymphocyte-associated protein 4, *IDO* Indoleamine 2,3-dioxygenase, *n* + Number of positive cases, + Positive; Combined n + , at least one of the immune checkpoints is positive; Red, significant difference (*p*-value ≤ 0.05); Blue, borderline significant difference (0.05 < *p*-value < 0.1).

**Table 3:**
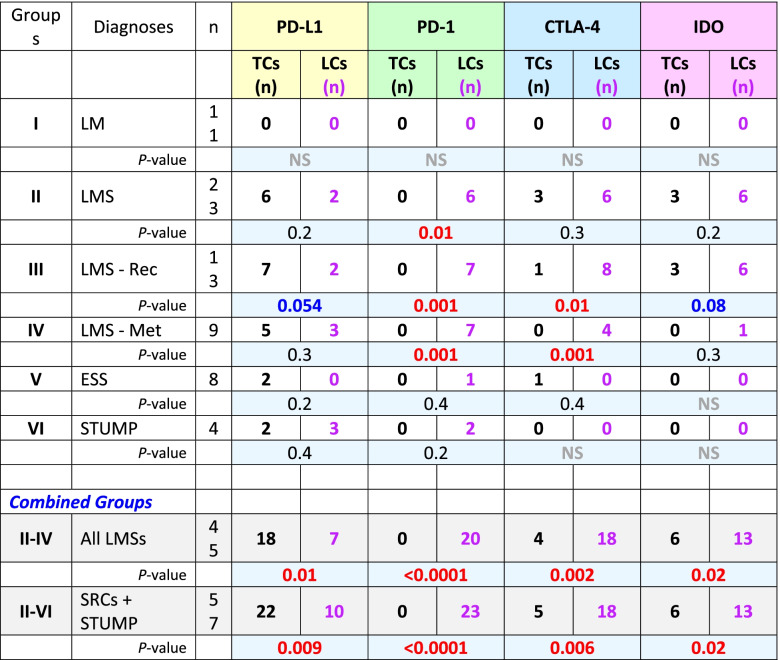
Student's T-test for the IHC reactive tumor cells versus their adjacent infiltrating lymphoid cell counterparts

*LM* Leiomyoma, *LMS* Leiomyosarcoma, *LMS—Rec* Recurrent leiomyosarcoma, *LMS—Met* Metastatic leiomyosarcoma, *EES* Endometrial stromal sarcoma, *STUMP* Smooth muscle tumor of uncertain malignant potential, *TC* Tumor cell, *LC* Lymphoid cell, *SRC* Sarcoma, *PD-L1* Programmed death-ligand 1, *PD-1* Programmed cell death protein 1, *CTLA-4* Cytotoxic T-lymphocyte-associated protein 4, *IDO* Indoleamine 2,3-dioxygenase, *NS* Total negative stain; Red, significant difference (*P-*values ≤ 0.05); Blue, borderline significant difference (0.05 *p-*value < 0.1).

**Table 4:**
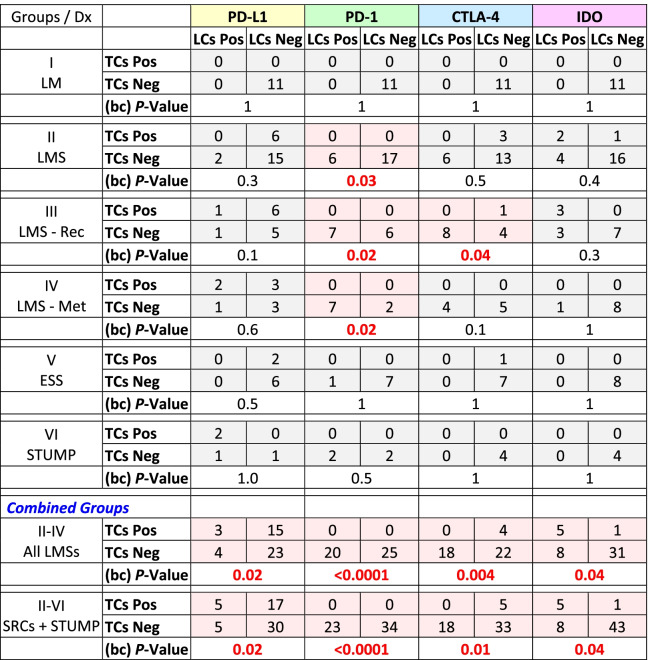
2 × 2 Tables of McNemar’s chi-square tests for matched-pairs tumor cells versus infiltrating lymphoid cells immunohistochemistry reactions, to compare proportionality

*Dx* Diagnosis, *LM* Leiomyoma, *LMS* Leiomyosarcoma, *LMS—Rec* Recurrent leiomyosarcoma, *LMS—Met* Metastatic leiomyosarcoma, *ESS* Endometrial stromal sarcoma, *STUMP* Smooth muscle tumor of uncertain malignant potential, *SRC* Sarcoma, *TCs* Tumor cells, *LCs* Lymphoid cells, *PD-L1* Programmed death-ligand 1, *PD-1* Programmed cell death protein 1, *CTLA-4* Cytotoxic T-lymphocyte-associated protein 4, *IDO* Indoleamine 2,3-dioxygenase, *Pos* Positive reaction, *Neg* Negative reaction; Red, significant difference (*p*-value ≤ 0.05)

## Results

During the period of 2005 to 2020, a total of 77 patients with mesenchymal tumors were identified by the computer search, of which, 68 were selected for this study. Among these patients, 11 had leiomyomas (Group I) and 57 had the sarcomas and STUMP (Groups II-VI). The cases, in the latter groups (II-VI), had a median age of 57 years while 20 were alive, 20 dead, and 17 had no follow-up at the time of this study. Due to a larger number of “no follow-ups”, it became implausible to correlate the immune reactions to the clinical outcome. However, the clinical status has been included in the tables for future meta-analyses.

Excluding the cases with leiomyoma, expression of at least one immune checkpoint had occurred in 28 of 57 (49%) tumors (Table 1) and 31 of 57 (54%) infiltrating lymphoid (Table 2) cells. In contrast, all 11 cases with leiomyoma (Group I) had neither lymphoid cell infiltrates and nor expression of any checkpoints (Additional Table 1). Comparing Group-I to the combined groups (II-IV & II-VI), *p*-values of < 0.0001 were rendered for expression of at least one immune checkpoint in tumor and/or infiltrating lymphoid cells (Tables 1 and 2). For individual immune checkpoints in the combined groups (II-VI), there were 22 (39%, PD-L1), 0 (0%, PD-1), 5 (9%, CTLA-4), and 6 (11%, IDO) cases who showed positive IHC staining in the tumor cells (Table 1). The majority of the of the positive reactions were detected in the cases with leiomyosarcoma (Groups II-IV); PD-L1 in 18 (40%), CTLA-4 in 4 (9%), and IDO in 6 (13%) cases (Table 1). Likewise, the lymphoid cells (Groups II-IV) were positive in 7 (16%, PD-L1), 20 (44%, PD-1), 18 (40%, CTLA-4), and 13 (29%, IDO) cases (Table 2). Using T-test, comparing Group-I versus Groups II-IV and II-VI for individual immune checkpoint, also resulted in *p*-values of 0.04 or less for the tumor (Table 1) as well as the lymphoid (Table 2) cells except for PD-1 where there were no reactions in the tumor cells (Table 1). The overall reactions for combined groups II-VI and the associated statistical analyses are listed in Tables 1 and 2.

In the combined groups (II-IV & II-VI), significant differences (*p*-values ≤ 0.02) were observed for all four checkpoints when their expressions in the tumor cells were compared to the lymphoid cell counterparts (Table 3). Similar values (*p*-values ≤ 0.04) were also observed when McNemar’s chi-square analyses were carried out for the proportional distributions (Table 4). These analyses indicate the expression of immune checkpoints between tumor cells and their immune cell counterparts are mutually exclusive. Cases-in-point is PD-L1 in Group II, where the checkpoint had been expressed in the tumor cells of 6 cases (case# 29–34, Additional Table 1) while the same protein showed expression in the lymphoid cells of two cases other than the 6 (case# 22 & 28, Additional Table 1). For each individual group refer to Table 3 for the Student’s T-test and Table 4 for McNemar’s chi-square test results.

### Group I, leiomyoma (LM)

Leiomyomas are the most common benign mesenchymal tumors of smooth muscle derivations in uterus [19]. There were 11 cases with a median age of 40 years in this group which were selected randomly from recent cases in our medical center, unlike the patients with sarcomas which were selected in totality within the specified period. None of these cases showed any expression of the four immune checkpoints and there were no lymphoid infiltrates in the lesions. This group served as the control for comparisons with the subsequent groups (Additional Table 1).

### Group II, leiomyosarcoma (LMS)

Leiomyosarcomas are the most common malignant uterine mesenchymal tumors that present in older aged adults and have an overall 5-year survival of approximately 15–25%. Microscopically, they are hypercellular and mitotically active with areas of nuclear atypia and necrosis [20]. There were 23 cases with LMS, originally detected in the uterus, who had a median age of 58 years.

**Fig. 3:**
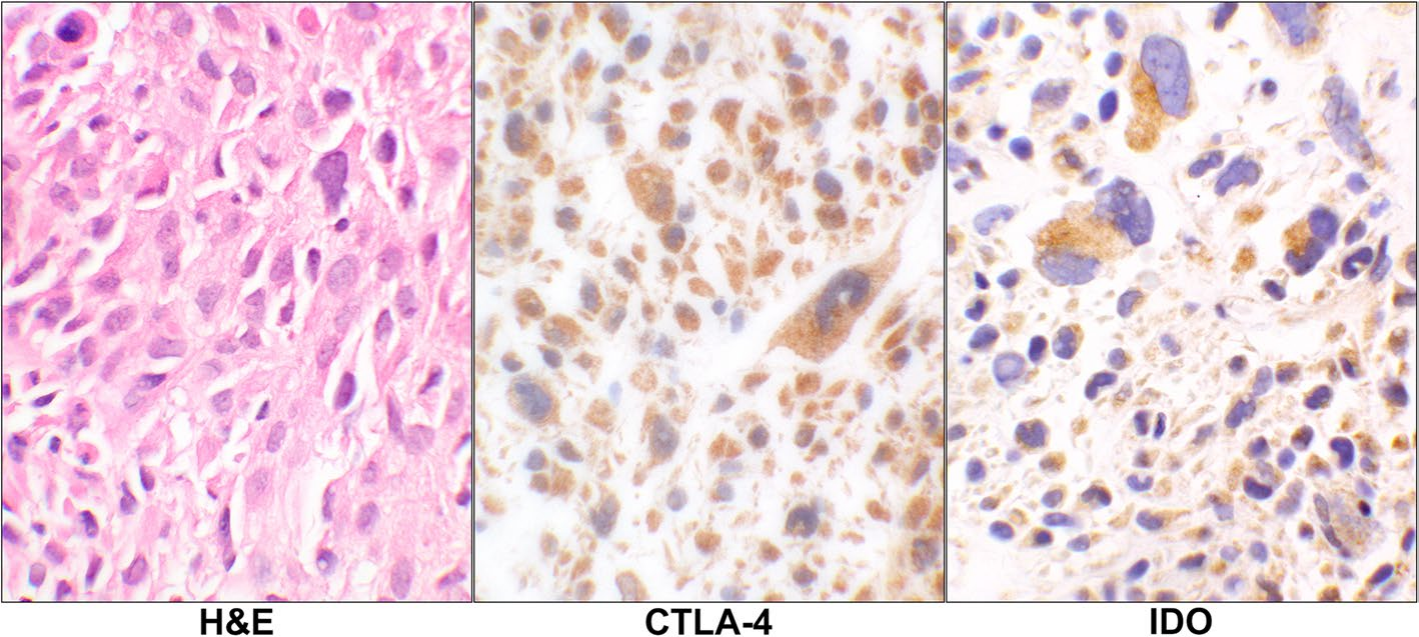
CTLA-4 and IDO reactions in Leiomyosarcoma. H&E stain of a case of primary leiomyosarcoma showing pleomorphic malignant cells (case# 17, Additional Table 1). The corresponding CTLA-4 and IDO IHC stains show positive granular cytoplasmic staining of the malignant cells. (40 × objective). Abbreviations: H&E, hematoxylin & eosin; CTLA-4, cytotoxic T lymphocyte antigen 4; IDO, indolaimine-2, 3-deoxygenase; IHC, immunohistochemistry

For the tumor cells, there were 6 (26%, PD-L1), 0 (0%, PD-1), 3 (13%, CTLA-4), and 3 (13%, IDO) cases showing IHCs positivity. An example of the latter two IHC reactions is displayed in Fig. 3. Overall, there were 10 cases (43%) showing expression for one or more of these immune checkpoints in tumor cells in this group (Table 1).

**Fig. 4:**
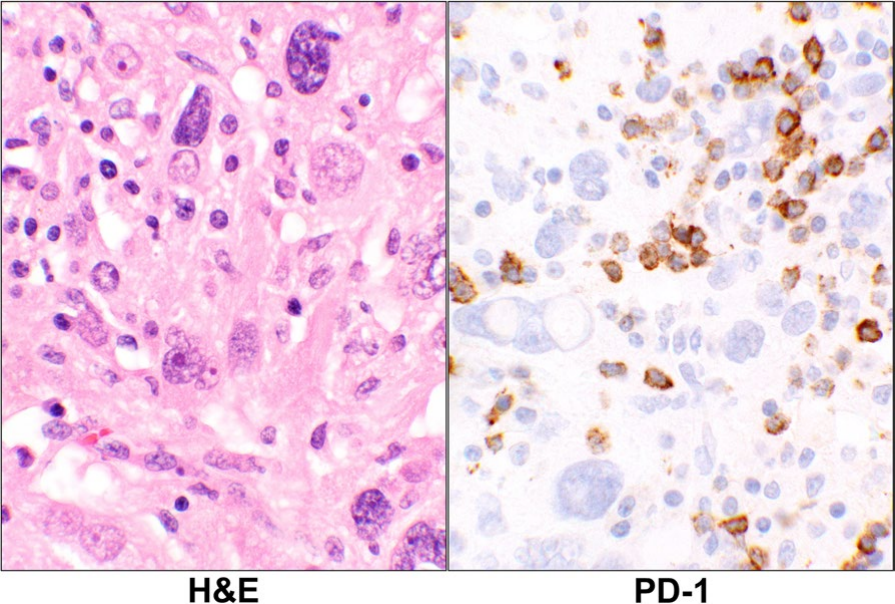
PD-1 Reaction in Leiomyosarcoma. H&E stain of a case with primary leiomyosarcoma (case# 28, Additional Table 1) showing large malignant cells and scattered small lymphoid cells in the background. The corresponding PD-1 stain shows a positive staining of some of the lymphoid cells while the tumor as well as the remaining lymphoid cells are negative (40 × objective). Abbreviations: H&E, hematoxylin & eosin; PD-1, programmed cell death protein 1

For the lymphoid cells, 2 (9%, PD-L1), 6 (26%, PD-1), 6 (26%, CTLA-4), and 6 (26%, IDO) cases showing IHC positivity. Overall, in 11 cases (48%) the lymphoid cells showed expression of one or more of the checkpoints (Additional Tables 1 and 2). An example of the lymphoid cells with PD-1 positivity is shown infiltrating the tumor cells (Fig. 4).

### Group III, leiomyosarcoma, recurrent (LMS-Rec)

There were 13 cases with a history of uterine leiomyosarcoma with a recurrent tumor as a pelvic or lower-abdominal mass. In this group, the median age was 52 years.

For the tumor cells, there were 7 (54%, PD-L1), 0 (0%, PD-1), 1 (8%, CTLA-4), 3 (23%, IDO) cases showed positive IHCs staining. Overall, 9 cases (69%) showed expression of one or more of the immune checkpoints in the tumor cells (Table 1).

For the lymphoid cells, 2 (15%, PD-L1), 7 (54%, PD-1), 8 (62%, CTLA-4), and 6 (46%, IDO) cases showed IHC positivity. Overall, in 9 cases (69%), the lymphoid cells showed expression of one or more of the immune checkpoint proteins (Table 2).

### Group IV, Leiomyosarcoma, metastatic (LMS-Met)

There were 9 cases with a median age of 50 years in this group. The site of metastases were femoral head (3), scapula (1), gluteal muscle (1), lung (2), brain (1), and groin (1).

**Fig. 5:**
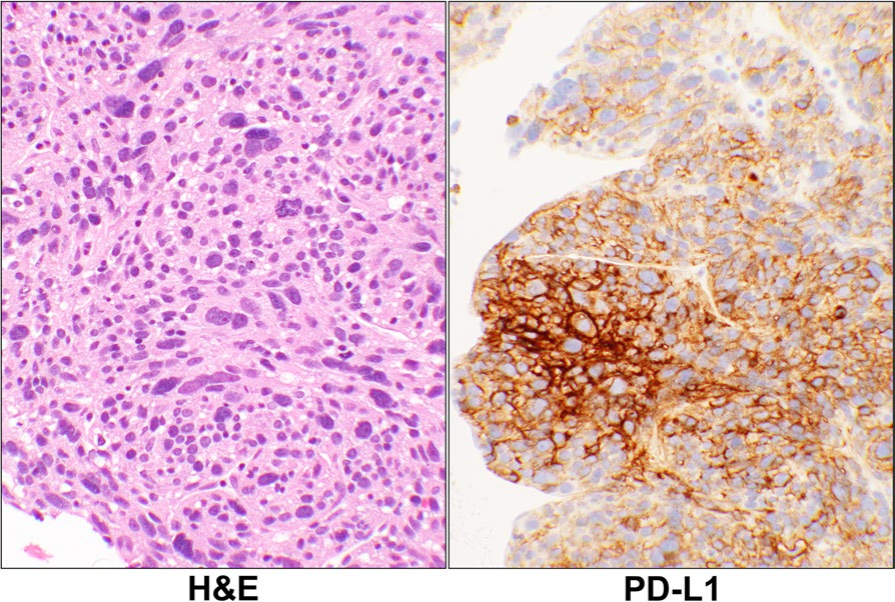
Metastatic Leiomyosarcoma. H&E stain of a case of metastatic leiomyosarcoma with pleomorphic cells (case# 57, Additional Table 1). The corresponding PD-L1 IHC stain showed 90% positivity of the tumor cells with an intensity of 3 + . (20 × objective). Abbreviations: H&E, hematoxylin & eosin; PD-L1, programmed death-ligand 1

For the tumor cells, there were 5 (56%, PD-L1), 0 (0%, PD-1), 0 (0%, CTLA-4), 0 (0%, IDO) cases showed positive IHCs staining (Fig. 5). There were no tumors showing expression of the immune checkpoints other than PD-L1 in this group (Additional Table 1 and Table 1).

For the lymphoid cells, 3 (33%, PD-L1), 7 (78%, PD-1), 4 (44%, CTLA-4), and 1 (11%, IDO) case showed IHC positivity for the proteins. Overall, the lymphoid cells showed expression for one or more of the checkpoint proteins in 8 (89%) cases (Additional Tables 1 and 2).

### Group V, endometrial stromal sarcoma (ESS)

Endometrial stromal tumors arise from the stromal cells of the endometrium which could be either endometrial stomal nodules (ESN) or ESS. *Endometrial stromal sarcomas* are the second most common malignant uterine mesenchymal neoplasms which are divided into low-grade and high-grade categories [43]. No ESN lesions were present in this series.

There were 8 cases with a primary diagnosis of ESS in uterus who had a median age of 48 years.

**Fig. 6:**
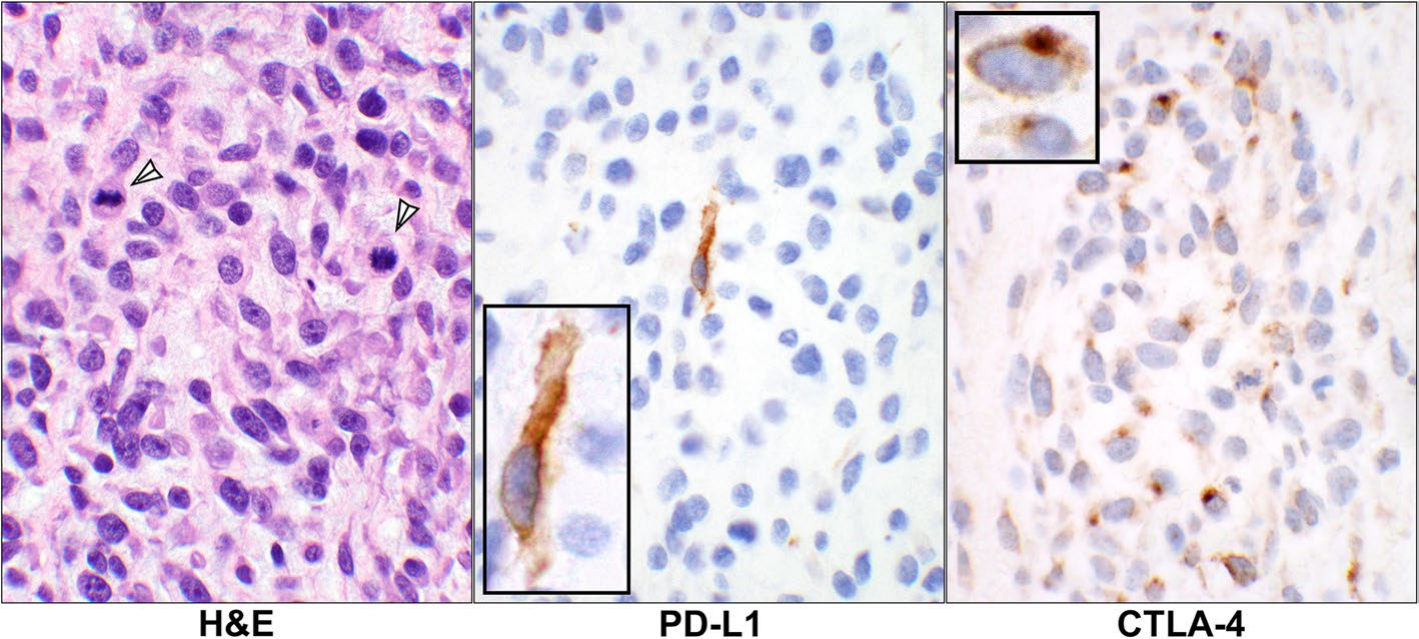
Staining reactions in endometrial stromal sarcoma. H&E stain of a case of a uterine endometrial stromal sarcoma showing unform malignant stromal cells containing mitotic figures (arrowheads). The corresponding PD-L1 stain shows a single tumor cell with the cell membrane reactivity and CTLA-4 IHC displaying positive granular cytoplasmic staining of the malignant cells which are very similar to that in the control tissue as seen in Fig. 1 (case# 63, Additional Table 1). (20 × objective, insets: 60 × objective). Abbreviations: H&E, hematoxylin & eosin; PD-L1, programmed death-ligand 1; CTLA-4, cytotoxic T lymphocyte antigen 4

For the tumor cells, there were 2 (25%, PD-L1), 0 (0%, PD-1), 1 (13%, CTLA-4), 0 (0%, IDO) cases which showed positive IHC staining (Fig. 6). Overall, 2 cases (25%) showed the expressions manifested by the antibodies in which one had positivity for PD-L1 and the other for both PD-L1 and CTLA-4 (Additional Table 1 and Table 1).

For the lymphoid cells, 0 (0%, PD-L1), 1 (13%, PD-1), 0 (0%, CTLA-4), and 0 (0%, IDO) cases showing IHC positivity for only 1 case (13%) in the lymphoid cells (Table 2).

### Group VI, smooth muscle tumors of uncertain malignancy (STUMP)

**Fig. 7:**
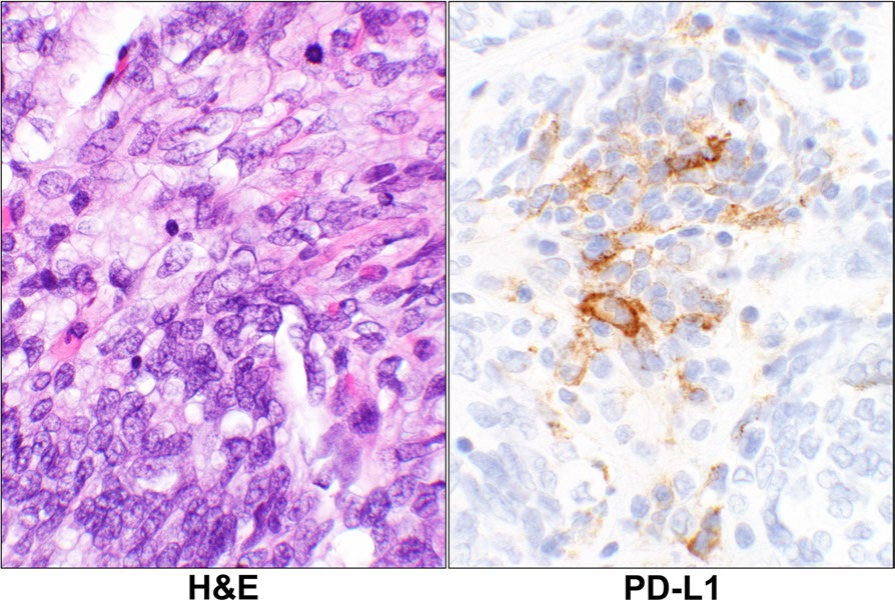
IHC reaction in STUMP. H&E stain of a case of smooth muscle tumor of low malignant potential with mildly atypical cells (case# 67, Additional Table 1). The corresponding PD-L1 stain showing focal cell membrane staining of the neoplastic cells. A single cell shows the strong cell membrane reaction. (40 × objective). Abbreviations: H&E, hematoxylin & eosin; PD-L1, programmed death-ligand 1

Smooth muscle tumors of uncertain malignant potential are the tumors that have some but not all the criteria of leiomyosarcoma [23]. There were 4 cases, originally detected in the uterus, who had a median age of 43 years in this group. For the tumor cells, there were only 2 (50%) cases who showed the IHC reaction for PD-L1 (Fig. 7). No IHC reactions were present for other checkpoints in the tumor cells (Additional Table 1 and Table 1).

For the lymphoid cells, 3 (75%, PD-L1), 2 (50%, PD-1), 0 (0%, CTLA-4), and 0 (0%, IDO) cases showing IHC positivity. Overall, in 3 cases (75%) the lymphoid cells showed expression of one or more of the immune checkpoints (Additional Tables 1 and 2).

## Discussion

While none of the 11 benign leiomyoma masses showed any expression of the check-point proteins in our study, nearly half (49%) of the uterine sarcomas and STUMP tumors collectively express at least one of the immune checkpoint molecules (Table 1). Specifically, 40% of leiomyosarcomas, 25% of endometrial stromal sarcomas, and 50% of STUMP tumors show PD-L1 immunoreactivity. Moreover, 9% and 13% of leiomyosarcomas express CTLA-4 and IDO proteins, respectively. The incidence of the expressions is not substantially different in primary, recurrent, and metastatic leiomyosarcomas. Interestingly, the infiltrating lymphoid cells have an opposite pattern of reactivity. Namely, when the tumor cells express an immune checkpoint, the adjacent infiltrating lymphoid cells do not and vice versa, shown by the Student’s T-test and McNemar’s chi-square tests (Tables 3 and 4). While PD-L1 is more expressed in the tumor cells, the expression of CTLA-4 and IDO conversely appear in the lymphoid cells except for PD-1 which is exclusively in the LCs. While the leiomyoma tumor cells have had neither check-point protein expressions nor any lymphoid cell infiltrates, 54% (31 of 57 patient) of the sarcomas and STUMP have harbored lymphoid tumor infiltrates with at least one check-point protein expression.

Uterine sarcomas account for 1% of gynecological cancers and 3–7% of all uterine malignancies [44]. Presently, uterine leiomyosarcomas and high grade endometrial stromal sarcomas remain unresponsive to available adjuvant radiation, chemotherapy, or hormonal treatments [2].

In the past decade, numerous studies have shown the effectiveness of immune checkpoint therapeutics in tackling several malignancies, particularly carcinomas [13, 14, 38, 45–48] and melanomas [16, 49, 50]. Several recent studies are actively investigating the potential role of targeted immunotherapy in a wide range of sarcomas [51]. However, their potential role in the fight against uterine sarcomas has not been thoroughly investigated. In a recent multicenter study, the investigators had used a cohort of 1115 subjects with leiomyosarcomas of which 701 were of uterine origin. In this study, the samples were subjected to next generation sequencing (NGS) of DNA and RNA to identify high tumor mutational burden (TMB) [6]. In addition, microsatellite instability (MSI) by IHC and NGS as well as PD-L1 staining by IHC were performed. The authors of this abstract have concluded that only a small proportion of LMSs are TMB-H or MSI-H, suggesting that the neoantigen burden in LMS may be insufficient to promote a robust anti-tumor response, even in the presence of PD-L1 positive tumor cells [6]. They further concluded that immune microenvironment of the LMS tumors is characterized by a low T cell abundance relative to melanoma. Although we did not stain the infiltrating lymphoid cells for CD8, our findings are in agreement with theirs. Namely, the IHC reactive lymphoid cells are absent particularly when the tumor cells express the corresponding immune checkpoint as found in this series (Table 4). Perhaps it can be postulated that presence of the IHC positivity of the *lymphoid cells* might be a better indicator of response to the treatment than expression of the immune checkpoints in the *tumor cells*, particularly PD-L1.

Here, we have demonstrated that a significant portion of malignant uterine sarcomas and their tumor-infiltrating inflammatory cells express targetable checkpoint proteins. Although our sample size is small due to the rare nature of the uterine mesenchymal tumors, a high percentage of the immune checkpoint’s expression has occurred in leiomyosarcomas. Therefore, our findings support the value of checkpoint inhibitor screening studies on post-surgical malignant uterine mesenchymal tumor specimens. The biomarkers from this study should be explored in future studies and may help select patients for immune checkpoint inhibitor therapy.

Overall, our findings are in line with the results of recent studies that found high PD1 and PD-L1 expression in soft tissue leiomyosarcomas [52] and PD-L1 expression in uterine leiomyosarcomas and their associated lymphocytic infiltrates [1]. Together, these findings provide support for screening checkpoint proteins, such as PD1, PD-L1, IDO, and CTLA-4 in post-surgical uterine sarcomas as they provide the means for the utilization of targeted immunotherapies in these rare but aggressive uterine sarcomas. Though this study supports testing the expression of checkpoint proteins in uterine malignant mesenchymal tumors, the clinical efficacy of the checkpoints blockade, individually or in combination, needs to be explored in clinical trials targeting these cancers.

## Supplementary Information

**Additional file 1: Table 1.** Summary of the immunohistochemical (IHC) reactions of the immune checkpoints in patients with uterine mesenchymal tumors.

**Additional file 2: Table 2.** Binary representation of positive (1) and negative (0) immunohistochemical (IHC) reactions of the immune checkpoints in patients with uterine mesenchymal tumors.
