# Supplementary material for: Expression of immune checkpoint regulators, programmed death-ligand 1 (PD-L1/PD-1), cytotoxic T lymphocyte antigen 4 (CTLA-4), and indolaimine-2, 3-deoxygenase (IDO) in uterine mesenchymal tumors
Source: Diagn Pathol. 2022 Sep 14;17:70. doi: 10.1186/s13000-022-01251-2 (PMC9476344; doi:10.1186/s13000-022-01251-2)
Supplement: Supplementary file 2 — Additional file 2: Table 2. Binary representation of positive (1) and negative (0) immunohistochemical (IHC) reactions of the immune checkpoints in patients with uterine mesenchymal tumors. [file 13000_2022_1251_MOESM2_ESM.pdf]

Additional Table 2. Binary representation of positive (1) and negative (0) immunohistochemical (IHC) reactions of the immune checkpoints in patients with uterine mesenchymal tumors

NO	Age	Diagnosis	PD-L1		PD-1		CTLA-4		IDO		Status	Combined	
			TCs	LCs	TCs	LCs	TCs	LCs	TCs	LCs		TCs	LCs
**Group-I (n = 11)**													
1	75	LM	0	0	0	0	0	0	0	0		0	0
2	40	LM	0	0	0	0	0	0	0	0		0	0
3	35	LM	0	0	0	0	0	0	0	0		0	0
4	33	LM	0	0	0	0	0	0	0	0		0	0
5	40	LM	0	0	0	0	0	0	0	0		0	0
6	40	LM	0	0	0	0	0	0	0	0		0	0
7	48	LM	0	0	0	0	0	0	0	0		0	0
8	40	LM	0	0	0	0	0	0	0	0		0	0
9	43	LM	0	0	0	0	0	0	0	0		0	0
10	34	LM	0	0	0	0	0	0	0	0		0	0
11	57	LM	0	0	0	0	0	0	0	0		0	0
**Group-II (n = 23)**													
12	68	LMS	0	0	0	0	0	1	1	1	NF	1	1
13	77	LMS	0	0	0	0	0	0	0	0	A	0	0
14	61	LMS	0	0	0	0	1	0	1	1	NF	1	1
15	67	LMS	0	0	0	0	0	0	0	0	D	0	0
16	41	LMS	0	0	0	0	0	0	0	0	D	0	0
17	72	LMS	0	0	0	0	1	0	1	0	D	1	0
18	37	LMS	0	0	0	0			0	0	NF	0	0
19	53	LMS	0	0	0	0	0	1	0	0	D	0	1
20	61	LMS	0	0	0	0	0	0	0	0	D	0	0
21	58	LMS	0	0	0	0	1	0	0	0	D	1	0
22	39	LMS	0	1	0	1	0	0	0	1	A	0	1
23	45	LMS	0	0	0	0	0	0	0	0	A	0	0
24	44	LMS	0	0	0	0	0	0	0	0	A	0	0
25	75	LMS	0	0	0	0	0	0	0	0	D	0	0
26	64	LMS	0	0	0	0	0	0	0	0	NF	0	0
27	43	LMS	0	0	0	0	0	0	0	0	NF	0	0
28	56	LMS	0	1	0	1	0	1	0	1	D	0	1
29	70	LMS	1	0	0	0	0	0	0	0	D	1	0
30	62	LMS	1	0	0	1	0	1	0	1	D	1	1
31	71	LMS	1	0	0	1	0	0	0	1	A	1	1
32	45	LMS	1	0	0	1	0	0	0	0	NF	1	1
33	49	LMS	1	0	0	0	0	1	0	0	NF	1	1
34	46	LMS	1	0	0	1	0	1	0	0	A	1	1
**Group-III (n = 13)**													
35	60	LMS - Rec	0	0	0	0	1	0	0	0	D	1	0
36	50	LMS - Rec	0	0	0	0	0	1	1	1	D	1	1
37	53	LMS - Rec	0	0	0	0	0	0	0	0	NF	0	0
38	52	LMS - Rec	0	0	0	0	0	0	0	0	D	0	0
40	49	LMS - Rec	0	0	0	1	0	0	0	0	A	0	1
41	54	LMS - Rec	0	1	0	1	0	1	0	1	A	0	1
42	49	LMS - Rec	1	0	0	1	0	1	0	1	NF	1	1
43	55	LMS - Rec	1	1	0	1	0	1	1	1	A	1	1
44	46	LMS - Rec	1	0	0	0	0	0	0	0	A	1	0
45	55	LMS - Rec	1	0	0	1	0	1	1	1	NF	1	1
46	51	LMS - Rec	1	0	0	0	0	1	0	1	D	1	1
47	79	LMS - Rec	1	0	0	1	0	1	0	0	A	1	1
48	48	LMS - Rec	1	0	0	1	0	1	0	0	A	1	1
**Group-IV (n = 9)**													
49	46	LMS - Met	0	1	0	1	0	1	0	0	A	0	1
50	68	LMS - Met	0	0	0	1	0	0	0	0	A	0	1
51	50	LMS - Met	0	0	0	0	0	0	0	0	D	0	0
52	50	LMS - Met	0	0	0	1	0	1	0	1	D	0	1
53	59	LMS - Met	1	0	0	1	0	0	0	0	A	1	1
54	50	LMS - Met	1	1	0	0	0	0	0	0	D	1	1
55	58	LMS - Met	1	1	0	1	0	0	0	0	D	1	1
56	47	LMS - Met	1	0	0	1	0	1	0	0	A	1	1
57	68	LMS - Met	1	0	0	1	0	1	0	0	NF	1	1
**Group-V (n = 8)**													
39	58	ESS, LG, Rec	0	0	0	0	0	0	0	0	A	0	0
58	46	ESS, HG	0	0	0	0	0	0	0	0	D	0	0
59	44	ESS, HG	0	0	0	0	0	0	0	0	A	0	0
60	60	ESS, HG	0	0	0	0	0	0	0	0	NF	0	0
61	42	ESS, HG	0	0	0	0	0	0	0	0	NF	0	0
62	48	ESS, HG	0	0	0	0	0	0	0	0	A	0	0
63	65	ESS, HG	1	0	0	0	1	0	0	0	D	1	0
64	74	ESS, HG	1	0	0	1	0	0	0	0	NF	1	1
**Group-VI (n = 4)**													
65	45	STUMP	0	0	0	0	0	0	0	0	NF	0	0
66	31	STUMP	0	1	0	0	0	0	0	0	NF	0	1
67	64	STUMP	1	1	0	1	0	0	0	0	NF	1	1
68	41	STUMP	1	1	0	1	0	0	0	0	A	1	1

LM, leiomyoma; **LMS**, leiomyosarcoma; **Rec**, recurrent; **Met**, metastatic leiomyosarcoma; **ESS**, endometrial stromal sarcoma; **LG**, low grade; **HG**, high grade; **STUMP**, smooth muscle tumor of uncertain malignant potential; **PD-L1**, programmed death-ligand 1; **PD-1**, programmed cell death protein 1; **CTLA-4**, cytotoxic T-lymphocyte-associated protein 4; **IDO**, indoleamine 2,3-dioxygenase; **TC**, tumor cell; **LC**, lymphoid cell; **A**, alive; **D**, dead; **NF**, no follow-up.
