# Supplementary material for: Expression of immune checkpoint regulators, programmed death-ligand 1 (PD-L1/PD-1), cytotoxic T lymphocyte antigen 4 (CTLA-4), and indolaimine-2, 3-deoxygenase (IDO) in uterine mesenchymal tumors
Source: Diagn Pathol. 2022 Sep 14;17:70. doi: 10.1186/s13000-022-01251-2 (PMC9476344; doi:10.1186/s13000-022-01251-2)
Supplement: Supplementary file 1 — Additional file 1: Table 1. Summary of the immunohistochemical (IHC) reactions of the immune checkpoints in patients with uterine mesenchymal tumors. [file 13000_2022_1251_MOESM1_ESM.pdf]

Additional Table 1. Summary of the immunohistochemical (IHC) reactions of the immune checkpoints in patients with uterine mesenchymal tumors

NO	Age	Diagnosis	PD-L1					PD-1					CTLA-4					IDO					Status	Combined	
			TCs			LCs		TCs			LCs		TCs			LCs		TCs			LCs			TCs	LCs
**Group-I (n = 11)**			Score	%	Int	%	Int	Score	%	Int	%	Int	Score	%	Int	%	Int	Score	%	Int	%	Int			
1	75	LM	0	0	0	0	0	0	0	0	0	0	0	0	0	0	0	0	0	0	0	0		N	N
2	40	LM	0	0	0	0	0	0	0	0	0	0	0	0	0	0	0	0	0	0	0	0		N	N
3	35	LM	0	0	0	0	0	0	0	0	0	0	0	0	0	0	0	0	0	0	0	0		N	N
4	33	LM	0	0	0	0	0	0	0	0	0	0	0	0	0	0	0	0	0	0	0	0		N	N
5	40	LM	0	0	0	0	0	0	0	0	0	0	0	0	0	0	0	0	0	0	0	0		N	N
6	40	LM	0	0	0	0	0	0	0	0	0	0	0	0	0	0	0	0	0	0	0	0		N	N
7	48	LM	0	0	0	0	0	0	0	0	0	0	0	0	0	0	0	0	0	0	0	0		N	N
8	40	LM	0	0	0	0	0	0	0	0	0	0	0	0	0	0	0	0	0	0	0	0		N	N
9	43	LM	0	0	0	0	0	0	0	0	0	0	0	0	0	0	0	0	0	0	0	0		N	N
10	34	LM	0	0	0	0	0	0	0	0	0	0	0	0	0	0	0	0	0	0	0	0		N	N
11	57	LM	0	0	0	0	0	0	0	0	0	0	0	0	0	0	0	0	0	0	0	0		N	N
**Group-II (n = 23)**																									
12	68	LMS	0	0	0	0	0	0	0	0	0	0	0	0	0	1	2+	1a	1	1+	5	3+	NF	P	P
13	77	LMS	0	0	0	0	0	0	0	0	0	0	0	0	0	0	0	0	0	0	0	0	A	N	N
14	61	LMS	0	0	0	0	0	0	0	0	0	0	1a	40	1+	0	0	1a	40	1+	5	1+	NF	P	P
15	67	LMS	0	0	0	0	0	0	0	0	0	0	0	0	0	0	0	0	0	0	0	0	D	N	N
16	41	LMS	0	0	0	0	0	0	0	0	0	0	0	0	0	0	0	0	0	0	0	0	D	N	N
17	72	LMS	0	0	0	0	0	0	0	0	0	0	2b	60	2+	0	0	1b	10	2+	0	0	D	P	N
18	37	LMS	0	0	0	0	0	0	0	0	0	0	NT	NT	NT	NT	NT	0	0	0	0	0	NF	N	N
19	53	LMS	0	0	0	0	0	0	0	0	0	0	0	0	0	1	2+	0	0	0	0	0	D	N	P
20	61	LMS	0	0	0	0	0	0	0	0	0	0	0	0	0	0	0	0	0	0	0	0	D	N	N
21	58	LMS	0	0	0	0	0	0	0	0	0	0	2b	90	1+	0	0	0	0	0	0	0	D	P	N
22	39	LMS	0	0	0	1	2+	0	0	0	10	3+	0	0	0	0	0	0	0	0	1	2+	A	N	P
23	45	LMS	0	0	0	0	0	0	0	0	0	0	0	0	0	0	0	0	0	0	0	0	A	N	N
24	44	LMS	0	0	0	0	0	0	0	0	0	0	0	0	0	0	0	0	0	0	0	0	A	N	N
25	75	LMS	0	0	0	0	0	0	0	0	0	0	0	0	0	0	0	0	0	0	0	0	D	N	N
26	64	LMS	0	0	0	0	0	0	0	0	0	0	0	0	0	0	0	0	0	0	0	0	NF	N	N
27	43	LMS	0	0	0	0	0	0	0	0	0	0	0	0	0	0	0	0	0	0	0	0	NF	N	N
28	56	LMS	0	0	0	40	3+	0	0	0	40	3+	0	0	0	10	2+	0	0	0	5	2+	D	N	P
29	70	LMS	1a	4	1+	0	0	0	0	0	0	0	0	0	0	0	0	0	0	0	0	0	D	P	N
30	62	LMS	1a	10	1+	0	0	0	0	0	5	1+	0	0	0	5	2+	0	0	0	5	2+	D	P	P
31	71	LMS	1a	10	1+	0	0	0	0	0	10	3+	0	0	0	0	0	0	0	0	1	2+	A	P	P
32	45	LMS	1a	10	1+	0	0	0	0	0	5	1+	0	0	0	0	0	0	0	0	0	0	NF	P	P
33	49	LMS	1a	10	1+	0	0	0	0	0	0	0	0	0	0	5	3+	0	0	0	0	0	NF	P	P
34	46	LMS	1b	2	2+	0	0	0	0	0	1	2+	0	0	0	5	2+	0	0	0	0	0	A	P	P
**Group-III (n = 13)**																									
35	60	LMS - Rec	0	0	0	0	0	0	0	0	0	0	2b	90	1+	0	0	0	0	0	0	0	D	P	N
36	50	LMS - Rec	0	0	0	0	0	0	0	0	0	0	0	0	0	5	3+	1b	5	2+	20	3+	D	P	P
37	53	LMS - Rec	0	0	0	0	0	0	0	0	0	0	0	0	0	0	0	0	0	0	0	0	NF	N	N
38	52	LMS - Rec	0	0	0	0	0	0	0	0	0	0	0	0	0	0	0	0	0	0	0	0	D	N	N
40	49	LMS - Rec	0	0	0	0	0	0	0	0	1	2+	0	0	0	0	0	0	0	0	0	0	A	N	P
41	54	LMS - Rec	0	0	0	2	3+	0	0	0	10	2+	0	0	0	5	2+	0	0	0	5	2+	A	N	P
42	49	LMS - Rec	1a	10	1+	0	0	0	0	0	20	2+	0	0	0	2	2+	0	0	0	2	2+	NF	P	P
43	55	LMS - Rec	1b	5	2+	10	3+	0	0	0	40	3+	0	0	0	10	2+	1a	5	1+	5	3+	A	P	P
44	46	LMS - Rec	1b	10	2+	0	0	0	0	0	0	0	0	0	0	0	0	0	0	0	0	0	A	P	N
45	55	LMS - Rec	1b	20	2+	0	0	0	0	0	20	2+	0	0	0	30	3+	1a	1	1+	50	3+	NF	P	P
46	51	LMS - Rec	1b	30	3+	0	0	0	0	0	0	0	0	0	0	10	3+	0	0	0	5	2+	D	P	P
47	79	LMS - Rec	1b	30	2+	0	0	0	0	0	40	3+	0	0	0	5	2+	0	0	0	0	0	A	P	P
48	48	LMS - Rec	1b	30	3+	0	0	0	0	0	5	2+	0	0	0	5	2+	0	0	0	0	0	A	P	P
**Group-IV (n = 9)**																									
49	46	LMS - Met	0	0	0	1	1+	0	0	0	1	2+	0	0	0	1	2+	0	0	0	0	0	A	N	P
50	68	LMS - Met	0	0	0	0	0	0	0	0	5	2+	0	0	0	0	0	0	0	0	0	0	A	N	P
51	50	LMS - Met	0	0	0	0	0	0	0	0	0	0	0	0	0	0	0	0	0	0	0	0	D	N	N
52	50	LMS - Met	0	0	0	0	0	0	0	0	5	2+	0	0	0	5	2+	0	0	0	5	2+	D	N	P
53	59	LMS - Met	1a	10	1+	0	0	0	0	0	5	2+	0	0	0	0	0	0	0	0	0	0	A	P	P
54	50	LMS - Met	1b	5	3+	5	2+	0	0	0	0	0	0	0	0	0	0	0	0	0	0	0	D	P	P
55	58	LMS - Met	1b	30	2+	5	2+	0	0	0	5	2+	0	0	0	0	0	0	0	0	0	0	D	P	P
56	47	LMS - Met	1b	30	3+	0	0	0	0	0	5	2+	0	0	0	5	3+	0	0	0	0	0	A	P	P
57	68	LMS - Met	2b	90	3+	0	0	0	0	0	20	2+	0	0	0	1	2+	0	0	0	0	0	NF	P	P
**Group-V (n = 8)**																									
39	58	ESS, LG, Rec	0	0	0	0	0	0	0	0	0	0	0	0	0	0	0	0	0	0	0	0	A	N	N
58	46	ESS, HG	0	0	0	0	0	0	0	0	0	0	0	0	0	0	0	0	0	0	0	0	D	N	N
59	44	ESS, HG	0	0	0	0	0	0	0	0	0	0	0	0	0	0	0	0	0	0	0	0	A	N	N
60	60	ESS, HG	0	0	0	0	0	0	0	0	0	0	0	0	0	0	0	0	0	0	0	0	NF	N	N
61	42	ESS, HG	0	0	0	0	0	0	0	0	0	0	0	0	0	0	0	0	0	0	0	0	NF	N	N
62	48	ESS, HG	0	0	0	0	0	0	0	0	0	0	0	0	0	0	0	0	0	0	0	0	A	N	N
63	65	ESS, HG	1b	2	2+	0	0	0	0	0	0	0	1b	30	3+	0	0	0	0	0	0	0	D	P	N
64	74	ESS, HG	1b	5	2+	0	0	0	0	0	3	3+	0	0	0	0	0	0	0	0	0	0	NF	P	P
**Group-VI (n = 4)**																									
65	45	STUMP	0	0	0	0	0	0	0	0	0	0	0	0	0	0	0	0	0	0	0	0	NF	N	N
66	31	STUMP	0	0	0	2	2+	0	0	0	0	0	0	0	0	0	0	0	0	0	0	0	NF	N	P
67	64	STUMP	1b	1	2+	5	2+	0	0	0	20	2+	0	0	0	0	0	0	0	0	0	0	NF	P	P
68	41	STUMP	1b	1	2+	5	2+	0	0	0	10	2+	0	0	0	0	0	0	0	0	0	0	A	P	P

LM, leiomyoma; **LMS**, leiomyosarcoma; **Rec**, recurrent; **Met**, metastatic leiomyosarcoma; **ESS**, endometrial stromal sarcoma; **LG**, low grade; **HG**, high grade; **STUMP**, smooth muscle tumor of uncertain malignant potential; **PD-L1**, programmed death-ligand 1; **PD-1**, programmed cell death protein 1; **CTLA-4**, cytotoxic T-lymphocyte-associated protein 4; **IDO**, indoleamine 2,3-dioxygenase; **TC**, tumor cell; **LC**, lymphoid cell; **Int**, intensity of IHC reaction; **1a**, <50% of tumor cells with an IHC intensity of 1+; **1b**, <50% of tumor cells with an IHC intensity of 2+ or 3+; **2b**, ≥50% of tumor cells with an IHC intensity of 2+ or 3+; **P**, if the cells are reactive for any of the four immune checkpoints; **N**, if the cells are not reactive for any of the four immune checkpoints; **NT**, no tissue present; **A**, alive; **D**, dead; **NF**, no follow-up.
